# Exploring the role of empathy in prolonged grief reactions to bereavement

**DOI:** 10.1038/s41598-023-34755-y

**Published:** 2023-05-10

**Authors:** Takuya Yoshiike, Francesco Benedetti, Yoshiya Moriguchi, Benedetta Vai, Veronica Aggio, Keiko Asano, Masaya Ito, Hiroki Ikeda, Hidefumi Ohmura, Motoyasu Honma, Naoto Yamada, Yoshiharu Kim, Satomi Nakajima, Kenichi Kuriyama

**Affiliations:** 1 Department of Sleep-Wake Disorders, National Institute of Mental Health, National Center of Neurology and Psychiatry, 4-1-1 Ogawahigashi, Kodaira, Tokyo 187-8553 Japan; 2 Psychiatry and Clinical Psychobiology, Division of Neuroscience, Scientific Institute Ospedale San Raffaele, Milan, Italy; 3 University Vita-Salute San Raffaele, Milan, Italy; 4 Department of Behavioral Medicine, National Institute of Mental Health, National Center of Neurology and Psychiatry, Kodaira, Japan; 5 Department of Human Sciences, Faculty of Human Sciences, Musashino University, Tokyo, Japan; 6 National Center for Cognitive Behavior Therapy and Research, National Center of Neurology and Psychiatry, Kodaira, Japan; 7 National Institute of Occupational Safety and Health, Japan Organization of Occupational Health and Safety, Kawasaki, Japan; 8 Department of Information Sciences, Faculty of Science and Technology, Tokyo University of Science, Noda, Japan; 9 Department of Physiology, Showa University School of Medicine, Tokyo, Japan; 10 Department of Psychiatry, Shiga University of Medical Science, Otsu, Japan

**Keywords:** Cognitive neuroscience, Emotion, Social behaviour, Social neuroscience, Stress and resilience, Anxiety, Depression, Post-traumatic stress disorder, Neurophysiology

## Abstract

Grief reactions to the bereavement of a close individual could involve empathy for pain, which is fundamental to social interaction. To explore whether grief symptoms interact with social relatedness to a person to whom one directs empathy to modulate the expression of empathy, we administered an empathy task to 28 bereaved adults during functional magnetic resonance imaging, in which participants were subliminally primed with facial stimuli (e.g., faces of their deceased or living relative, or a stranger), each immediately followed by a visual pain stimulus. Individuals’ grief severity promoted empathy for the pain stimulus primed with the deceased’s face, while it diminished the neural response to the pain stimulus primed with the face of either their living relative or a stranger in the medial frontal cortex (e.g., the right dorsal anterior cingulate cortex). Moreover, preliminary analyses showed that while the behavioral empathic response was promoted by the component of “longing” in the deceased priming condition, the neural empathic response was diminished by the component of “avoidance” in the stranger priming condition. Our results suggest an association between grief reactions to bereavement and empathy, in which grief symptoms interact with interpersonal factors to promote or diminish empathic responses to others’ pain.

## Introduction

The death of a close other or a group member can cause intense behavioral reactions in both humans and nonhuman animals^1^. In humans, bereavement is known to increase acute mortality risk and also impact long-term mental health outcomes^2^. Most bereaved individuals adapt to this loss. However, one out of 10 bereaved adults may fail to integrate the loss into their ongoing lives^3^, resulting in prolonged grief disorder, which is characterized by an intense yearning that persists a year or more after the loss^4^. Despite the recent inclusion of this condition in the International Classification of Diseases 11th Revision (ICD-11) and Diagnostic and Statistical Manual of Mental Disorders, fifth edition, text revision (DSM-5-TR), limited knowledge about the behavioral and neural consequences of bereavement has hindered the mechanistic understanding of prolonged grief.

Empathy—feeling what another feels or knowing what another knows—is fundamental to the formation and maintenance of social bonding^5^. There is evidence that empathy is positively associated with prosocial behavior, that is, social behaviors intended to benefit others^6–8^. Since the bereavement of a close other represents a disruption of the social bond with that person, it can be assumed that grief reactions to bereavement influence the regulation of empathy among bereaved individuals in later life^9^. However, no objective explanation has clarified whether and how bereavement alters empathy.

If empathy is considered a trait, highly empathic individuals may be more likely to act to relieve another’s distress than those who are less empathic. However, even highly empathic individuals may not always be empathic, and the expression of empathy can vary within individuals in different situations of daily life^10,11^. In line with these observations in real-life situations, experimental evidence has accumulated that an individual expresses more empathy to another with a similar social background than to one with a different background, indicating the importance of social relatedness in the expression of empathy. For instance, how and which empathy networks are recruited when observing pain in another may vary depending on whether the pain receiver is a close friend of^12–14^ or the same-race with^15^ the observer. In addition, animal studies have shown that the strength of pain or fear that a mouse exhibits when observing another experiencing pain or fear was increased when these two mice were socially related, such as cage mates, but not when they were strangers to one another^16,17^. These findings both provide an opportunity to reconsider empathy as a state and emphasize the importance of interpersonal factors in the regulation of empathy^18,19^.

The available evidence from empirical and clinical studies suggests that bereaved individuals with prolonged grief continue to feel close to the deceased after the loss in relation to the altered neurobiological basis of attachment bonds (e.g., oxytocin system)^20–22^. Such bereaved individuals may in turn continue to feel detached or separated from other people in the real world^23–25^. Therefore, it is likely that while bereaved individuals maintain empathy for the deceased person for a long time, they have difficulty regulating empathy toward their living family members and/or unrelated others following bereavement. Hence, we hypothesize that prolonged grief can be considered a mental condition, in which social cues could differentially influence the behavioral and neural bases of empathy (e.g., enhanced expression of empathy in the deceased-related context). The dorsal anterior cingulate cortex (dACC) and adjacent areas (e.g., supplementary motor area, SMA), which are known to affect the organization of responses to observing pain in others, could play a role in promoting or diminishing the expression of empathy according to the social context^26,27^.

To explore the role of empathy in grief reactions to bereavement, we conducted behavioral and functional magnetic resonance imaging (fMRI) experiments in which each participant was subliminally exposed to priming faces, each representing a different social relationship with the participant [e.g., “deceased (DEC)” or “living (LIV)” relative, or “stranger (STR)”]. Each of these priming stimuli was followed by the presentation of an image depicting a hand that had received physical pain. Participants were then asked to rate the consequences of the pain observation during fMRI scanning. We included participants who had been bereaved for more than 12 months to focus on the prolonged behavioral and neural consequences of bereavement.

## Results

### Participants’ characteristics

Table 1 presents the characteristics of the 28 participants and their deceased relatives. The violent or sudden deaths of their deceased relatives were caused by murder (*n* = 6), accidents (*n* = 5), disasters (*n* = 1), or myocardial infarction (*n* = 1). No participants were diagnosed with current major depressive disorder or posttraumatic stress disorder (PTSD), but a participant was diagnosed with current dysthymia, while two participants were diagnosed with past major depressive disorder and one with past PTSD.

**Table 1:** Characteristics of participants and death events.

Characteristic	Whole sample
	(*n* = 28)
Age, years	49.5 (10.8)
Men/women, *n*	2/26
Right-handed, *n* (%)	26 (92.9)
Education, years	13.8 (1.4)
Time since loss, years	8.5 (9.2)
Age of person who died, years	55.3 (27.2)
Child or spouse of the bereaved, *n* (%)	11 (39.3)
Sudden or violent loss, *n* (%)	14 (50.0)
Grief symptoms, ICG score	19.8 (13.1)
Depression symptoms, BDI-II score	10.7 (7.7)
Posttraumatic stress symptoms, IES-R score	14.5 (14.6)
Psychiatric medication use, *n* (%)	5 (17.9)

Values are given as means (SD) except where noted.

BDI-II, Beck Depression Inventory-II; ICG, Inventory of Complicated Grief; IES-R, Impact of Event Scale-Revised.

### Post-scan detection task

As predicted, the results of the post-scan task confirmed that none of the participants were able to accurately distinguish between the DEC or LIV faces among the presented faces, indicating that the masked priming faces successfully served as a subliminal stimulus in the face–empathy task. Additional details are provided in the Supplementary material.

### Face task

As also predicted, there were no significant effects of the priming conditions on neural response, indicating that the subliminal presentation of the DEC or LIV faces had individually no significant effects on any known pain empathy regions. Further details are provided in the Supplementary material.

### Face–empathy task

#### Behavior

**Figure 1:**
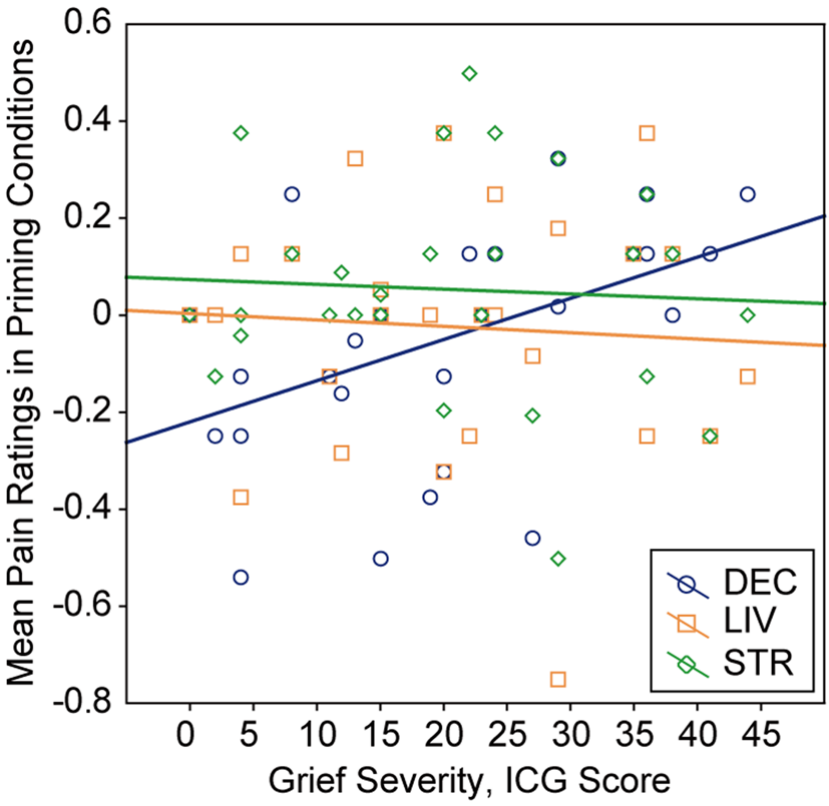
Interaction effects of individuals’ grief severity with the priming face conditions on pain ratings. The association between grief severity and pain ratings significantly differed according to the priming face conditions. Grief severity significantly influenced the pain ratings in the deceased (DEC) face condition, but not the living (LIV) or stranger (STR) face condition. The mean of pain ratings across trials in each priming face condition is shown as [DEC, LIV, or STR (painful > non-painful) > mosaic (painful > non-painful)].

We found that the more intensely participants were grieving in daily life, the more intensely they felt the pain stimulus, subliminally primed with their deceased’s face, as painful, independently of their comorbid psychological states (e.g., depression, posttraumatic stress symptoms). An analysis of variance (ANOVA) showed the differential patterns of association between grief severity and pain ratings across the priming face conditions (face × grief interaction: *F*_2,52_ = 4.11, *p* = 0.022, *η*^2^ = 0.137) (Fig. 1). Univariate tests showed that grief severity significantly influenced the pain ratings in DEC (*F*_1,26_ = 7.35, *p* = 0.012), but not the pain ratings in LIV (*F*_1,26_ = 0.13, *p* = 0.73) or STR (*F*_1,26_ = 0.09, *p* = 0.76). Individuals’ grief levels were positively correlated with the pain ratings in DEC (*r* = 0.47, *p* = 0.012), but not with those in LIV (*r* = − 0.07, *p* = 0.73) or STR (*r* = − 0.06, *p* = 0.76), the latter two of which, however, were moderately correlated with one another (*r* = 0.58, *p* = 0.001). A multiple regression showed that grief severity was an independent positive predictor of the pain ratings in DEC (log-likelihood = 4.65, *χ*^2^ = 6.97, *p* = 0.008). No significant effects were found for the severities of posttraumatic stress symptoms (log-likelihood = 4.76, *χ*^2^ = 0.19, *p* = 0.66) or depression symptoms (log-likelihood = 4.67, *χ*^2^ = 0.02, *p* = 0.87).

#### fMRI

**Figure 2:**
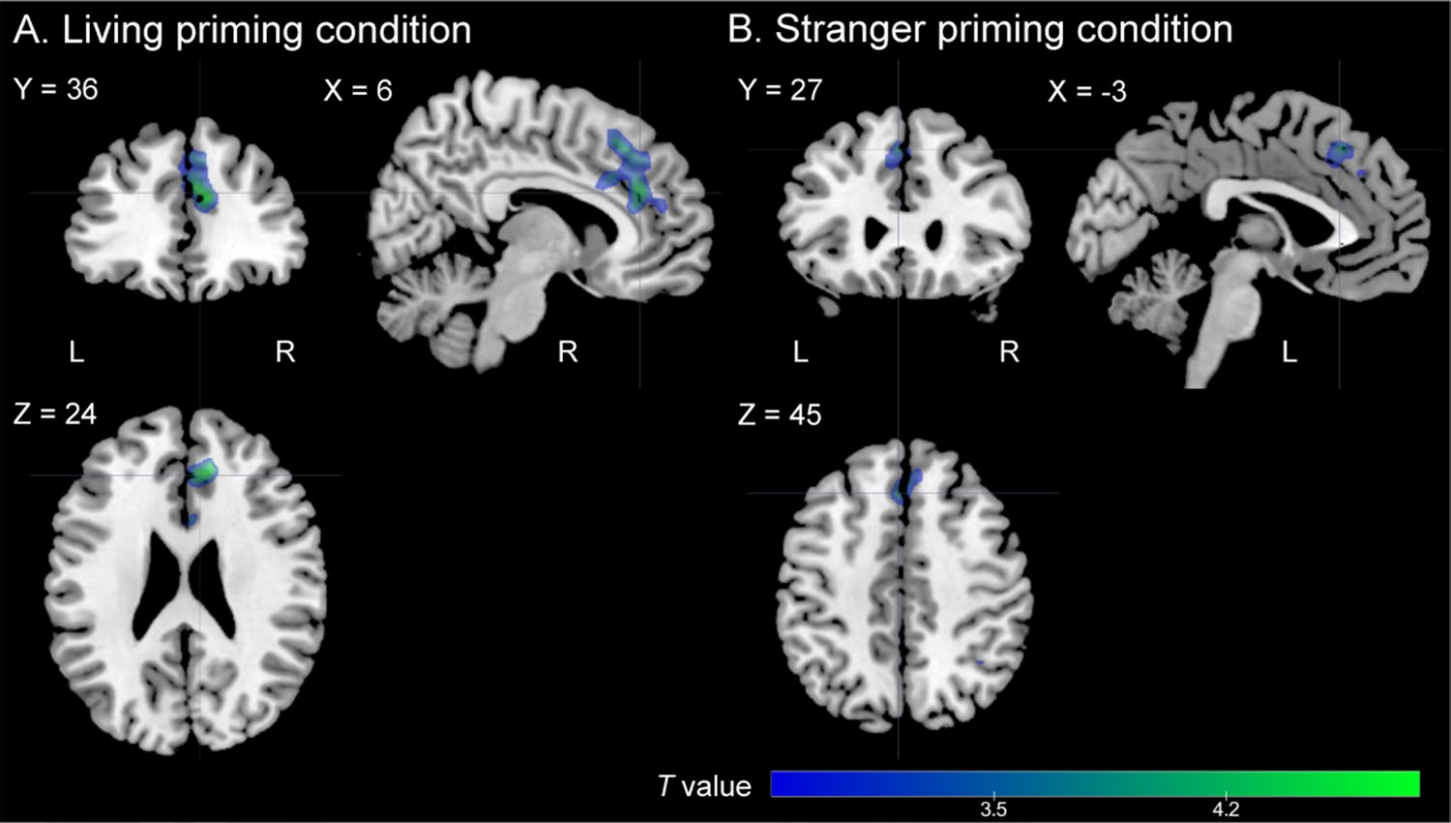
Significant clusters of correlation between grief severity and neural response during pain ratings. (**A**) In the living face condition, individuals’ grief severity was negatively correlated with neural response in a cluster with peak coordinates in the right dorsal anterior cingulate cortex within the identified pain empathy regions. (**B**) In the stranger face condition, the individual’s grief severity was also negatively correlated with neural response in another cluster with peak coordinates in the left superior medial frontal gyrus within the identified pain empathy regions. Clusters are shown in blue/green with *T*-values, and familywise error whole-brain corrected at *p* < 0.05.

We found that the more intensely participants were grieving in daily life, the less the task-related neural response in LIV and STR; however, this was not the case in DEC. We first identified the “pain empathy region” for the subsequent analyses. We observed significantly higher neural activation in four clusters, of which the strongest effect was found in a cluster across the bilateral superior medial frontal gyrus (SMFG), dACC, and SMA, during pain ratings in the painful condition than in the non-painful condition across all the priming conditions (Table S1; Fig. S1, Supplementary material). A multiple regression, with the neural response in LIV as the dependent variable, showed a significant negative effect of grief severity in a right-hemisphere dominant cluster across the bilateral dACC, SMFG, and right SMA with peak coordinates in the right dACC [Brodmann area 32, (6, 36, 24), *T*_1,21_ = 4.78, *Z* = 3.89, *p*_FWE_ = 0.002, *k* = 143; Fig. 2A] within the pain empathy regions. In addition, a multiple regression, with the neural response in STR as the dependent variable, showed a significant negative effect of grief severity in a left-hemisphere dominant cluster across the bilateral SMFG, dACC, and SMA with peak coordinates in the left SMFG [Brodmann area 8, (− 3, 27, 45), *T*_1,21_ = 4.13, *Z* = 3.49, *p*_FWE_ = 0.021, *k* = 94; Fig. 2B] within the pain empathy regions. However, a multiple regression, with the neural response in DEC as the dependent variable, showed no significant effect (*p*_FWE_ = 1.00) (Table 2).

**Table 2:** Brain areas in which grief severity was correlated with the neural response to pain ratings in each priming condition.

Priming condition	Hem	Brain area	BA	MNI coordinates			*T*	*Z*	*p*_FWE_	Cluster
Direction of effect				*x*	*y*	*z*				*k*
DEC										
Positive		No clusters								
Negative		No clusters								
LIV										
Positive		No clusters								
Negative	R	dACC	32	6	36	24	4.78	3.89	0.002	143
STR										
Positive		No clusters								
Negative	L	SMFG	6	− 3	27	45	4.13	3.49	0.021	94

Clusters shown are familywise error (FWE) corrected for multiple comparisons at *p* < .05.

BA, Brodmann area; dACC, dorsal anterior cingulate cortex; DEC, deceased relative; Hem, hemisphere; L, left; LIV, living relative; MNI, Montreal Neurological Institute; R, right; SMFG, superior medial frontal gyrus; STR, stranger.

### Association between the grief dimensions and empathy variables

We performed preliminary analyses to gain insights into which grief dimensions and behavioral/neural empathy variables were more influential on the association between grief and empathy. A principal component analysis (PCA) identified three significant dimensions of grief symptoms that explained 69.3% of the total variance of the scores (*Q*^2^ = 0.38): the first principal component (PC) involving most grief symptom items with the highest loading on “longing for the deceased”; the second PC mainly involving “hallucinations (auditory, visual),” and “physical pain”; and the third PC mainly involving “avoidance of loss reminders,” as well as “envy of others who have not lost someone” and “bitterness over the death” (Table S2, Supplementary material). An ANOVA with the pain ratings in DEC as the dependent variable, and with the three grief dimensions (PC scores) as continuous predictors showed a significant main effect of the first PC (*longing*:* F*_1,24_ = 6.79, *p* = 0.015, *η*^2^ = 0.221) but not of the second (*F*_1,24_ = 0.19, *p* = 0.67, *η*^2^ = 0.008) or third PC (*F*_1,24_ = 0.43, *p* = 0.52, *η*^2^ = 0.018). An ANOVA with the neural responses in LIV and STR as the dependent variables, the priming condition as the within-subjects factor (two levels), and the three grief dimensions (PC scores) as the continuous predictors showed a significant face × grief dimension interaction (*F*_2,24_ = 8.93, *p* = 0.006, *η*^2^ = 0.271). Univariate tests showed that the third PC significantly influenced the neural response in STR (*avoidance*:* F*_1,24_ = 5.37, *p* = 0.029) but not the first (*F*_1,24_ = 0.60, *p* = 0.45) or second PC (*F*_1,24_ = 1.38, *p* = 0.25). Although there were no significant effects of grief dimensions on the neural response in LIV (*F*_1,24_ < 2.90, *p* > 0.10), a strong positive correlation was found between the extracted neural response in LIV and that in STR (*r* = 0.97, *p* < 0.001). Therefore, the neural response in LIV was entered into the subsequent canonical correlation analysis (CCA).

**Figure 3:**
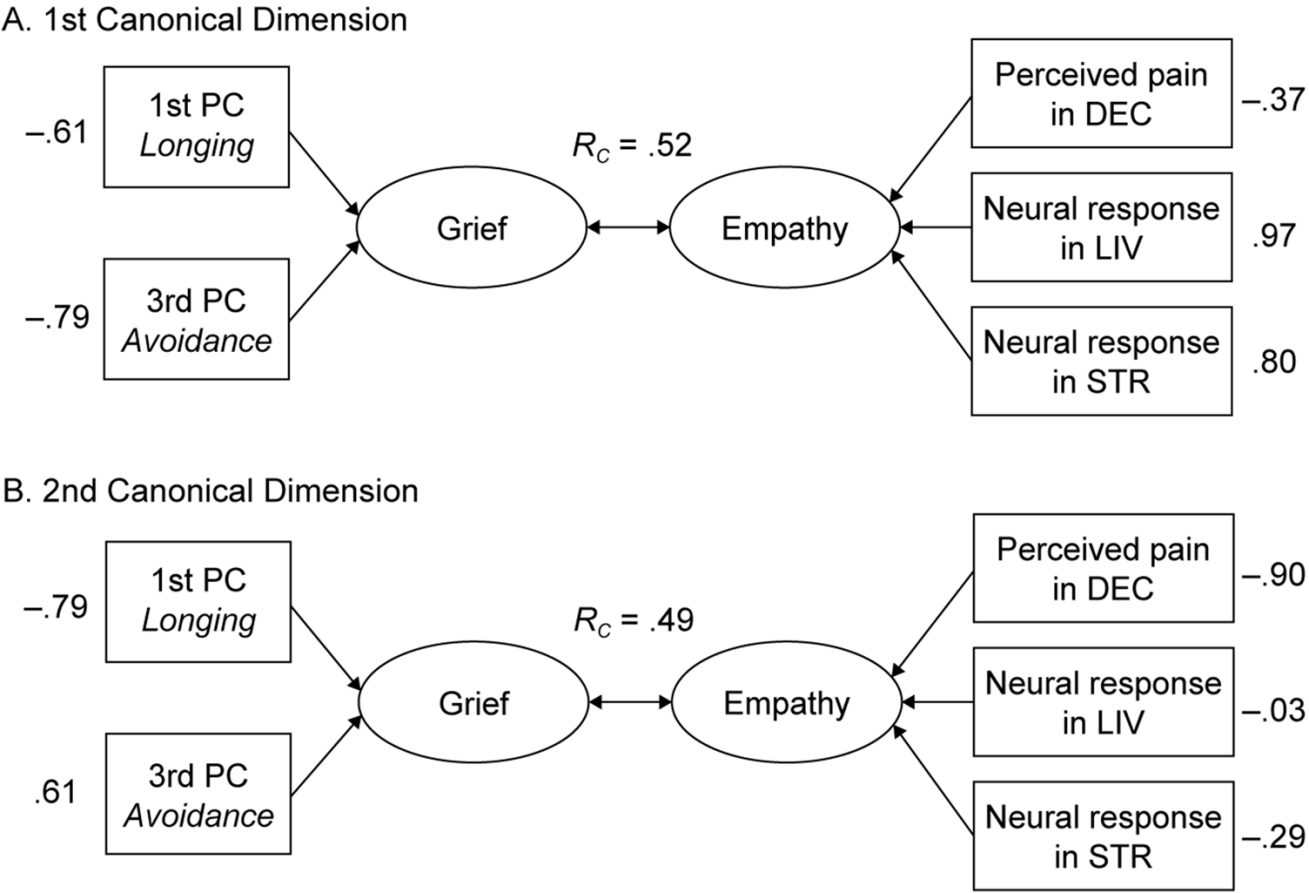
Diagrams depicting the results of the canonical correlation analysis. Two sets of variables, that is, grief variables (X) and empathy variables (Y), were moderately correlated with one another in two significant canonical roots. (**A**) The first canonical root, which explained more variance of the association between X and Y than the second canonical root, was most strongly influenced by the negative correlations of the third principal component (PC) of grief (loss-related avoidance) with the neural responses in the stranger (STR) and living (LIV) priming conditions. (**B**) The second canonical root was most strongly influenced by the positive correlation of the first PC of grief (longing for the deceased) with the pain ratings in the deceased (DEC) priming condition. The rectangles on the left and right denote the X and Y variables, respectively. The ovals denote the created synthetic variables. The values outside the rectangles denote the structure coefficients.

A CCA yielded two canonical roots. The full model was statistically significant (*p* = 0.005). Both the canonical roots significantly explained the total variance between the two variable sets (first pair: *R*_c_ = 0.63, *R*_c_^2^ = 0.39, *df* = 6, χ^2^ = 18.4, *p* = 0.005, Wilks λ = 0.46; second pair: *R*_c_ = 0.48, *R*_c_^2^ = 0.23, *df* = 2, χ^2^ = 6.38, *p* = 0.041, Wilks λ = 0.77). The first pair of canonical variates was most strongly influenced by the negative correlations between the third PC (*avoidance*) and neural response in STR and between the third PC (*avoidance*) and neural response in LIV (Fig. 3A), whereas the second pair of canonical variates was most strongly influenced by the positive correlation between the first PC (*longing*) and pain ratings in DEC (Fig. 3B).

## Discussion

The study results supported our hypothesis that empathy played a role in grief reactions to bereavement. As predicted, grief severity influenced the expression of empathy for pain, and the directions of the effects differed depending on the type of priming facial stimulus in our sample: grief severity promoted the behavioral empathic response to the pain stimulus subliminally associated with their deceased relative, while diminishing the neural empathic response to the pain stimulus subliminally associated with their living relative. No significant effects of comorbid posttraumatic stress or depressive symptoms on empathic responses were shown, supporting the specificity of the effects of grief symptoms on empathic expression in bereaved adults.

We predicted that bereaved individuals with higher grief severity would express higher empathy for the pain stimulus primed with their deceased relative than for that primed with their living relative or a stranger. Consistent with our prediction, individuals’ grief severity positively influenced their behavioral empathic response to the pain stimulus subliminally primed with the deceased’s face, but not with the faces of their living relative or a stranger. The deceased’s face itself, however, did not individually affect the brain’s empathy networks (as shown in the face task), indicating that the reminder of the deceased promoted empathy for pain through subliminal priming^28,29^. Moreover, our preliminary analyses showed that this positive effect of grief on empathy in the deceased priming condition was mostly explained by the grief component representing longing for the deceased. These findings are partly consistent with prior evidence that the social relatedness to an individual could influence the extent to which one expresses empathy to that individual^12–14^, and further suggests that even the subliminal presentation of social stimuli (e.g., priming faces with different social attributes) could induce a similar variability in the expression of empathy within individuals. Furthermore, our findings appear consistent with the clinical observation that bereaved individuals continue to feel close to their deceased person^30^, and with the reported alterations in reward networks^21^ or oxytocin regulation^20,22^, which underly human attachment behavior^9^.

Contrary to our prediction, however, participants’ grief severity did not increase the empathy-related neural response in the deceased priming condition. Although we could not provide a conclusive explanation for this null finding, one possible explanation is that among individuals who integrated the loss into their ongoing lives, empathic expressions were adequately weakened at the behavioral level but maintained implicitly at the neural level. Interestingly, we showed that individuals’ grief severity suppressed empathy-related neural activity when participants were exposed to living- or stranger-related stimuli, but not when exposed to deceased-related stimuli. This finding indicated that bereaved individuals with higher grief severity could have difficulty expressing empathy for pain when exposed to living- or stranger-related stimuli in daily life.

The brain areas, in which we observed the significant effects of an individual’s grief severity on the brain’s empathic response, were anatomically similar between the living and stranger priming conditions, with peak coordinates in the right dACC and left SMFG, respectively, and confined to the dACC, SMA, and SMFG. Multiple lines of evidence indicated that the dACC and adjacent areas (e.g., SMA) in the medial frontal cortex played an important role in processing information about others’ affective states, specifically related to pain (empathy for pain)^26,31–34^. These areas are further recruited to determine whether an action is needed and mediate the potential action according to given social contexts^12,27^. Although the exact role of the dACC in prolonged grief is unknown, neuroimaging evidence suggests that grief elicitation using loss reminders activates pain networks including the dACC^35,36^. Interestingly, albeit preliminary, our principal component analysis showed that increased loss-related avoidance (the third grief component) was associated with decreased activation in the right dACC in the stranger priming condition, suggesting that avoiding the painful reality of death could in turn limit the ability to empathize with others in daily life.

Importantly, there were notable similarities in empathic responses between the living and stranger priming conditions at both the behavioral and the neural levels. This suggested that bereaved individuals with increased grief severity may feel distant or separated from their living relatives at the subconscious or neural levels, as they should feel distant from a stranger. This finding provides a framework for understanding how bereavement can change the social location of their living relative between the deceased relative and unrelated others.

In our preliminary analyses, we found that the negative association of loss-related avoidance (the third grief component) with the stranger- and living-related empathic response explained more largely the grief–empathy relationship than did the positive association between longing for the deceased (the first grief component) and the deceased-related empathic response. Despite the exploratory nature of this part of the analysis, this preliminary finding could suggest that grief-related alterations in empathy could be more influenced by loss-related avoidance than longing for the deceased itself and raises questions about which components of grief are more important to maintain the consequences of the bereavement of a close other (e.g., longing vs. avoidance). This loss-related avoidance could then interfere with bereaved individuals’ ability to empathize with others in their later life. Avoidance of loss reminders, which is listed as a symptom of prolonged grief disorder in the DSM-5-TR, has been understood to be a behavioral construct that is difficult to assess as bereaved individuals are often unaware of things they avoid^37^. Persistent avoidance, however, may prolong grief processes as this behavioral strategy may reflect an attempt to avoid confronting the painful reality of the death^38^. Therefore, treating loss-related avoidance is a core element of psychotherapy for prolonged grief^39^. Future studies may examine whether reducing loss-related avoidance by facilitating both loss-related strategies (e.g., dealing with emotional pain) and restoration-related strategies (e.g., dealing with work, legal, and financial issues and taking on new roles), promotes adjustment for optimizing the reactivity of the core empathy network (e.g., dACC) to living- or stranger-related stimuli.

This study had several limitations. First, we employed a dimensional approach but did not employ any categorical criteria for this condition (e.g., prolonged grief disorder in the DSM-5-TR), because at the time we conducted this experiment, the diagnostic criteria had not been established, and it was common to determine individual’s grief severity using an instrument such as the ICG. Therefore, caution should be exercised when applying our data to the categorical understanding of prolonged grief. However, prolonged grief disorder can be viewed as the end of a spectrum of grief reactions to bereavement. Therefore, our findings may have clinical relevance for the mechanistic understanding of prolonged grief. Future studies need to elucidate the effects of prolonged grief on the regulation of empathy using a rating scale consistent with current diagnostic criteria such as the Prolonged Grief Disorder-13^40^. Second, although the two relationships between participants with their deceased and living relatives were considered similar by the participants, we did not quantify the extent to which participants felt close to their two relatives. Therefore, we cannot eliminate the possibility that the difference in the perceived closeness between their two relatives influenced our results. Third, in the current experimental paradigm, the hands that we associated with the reminders (i.e., faces) of participants’ deceased and living relatives were anonymous ones, which were not exactly the same as the hands of their deceased and living relatives, because we intended to prevent participants from changing their ratings of perceived pain intensity according to whether a presented hand belonged to their deceased or living relatives. However, a person’s identity is often perceived from facial cues rather than morphological features of the rest of the body including a hand^41^. In addition to this attentional priority of facial cues, hand ownership can be flexibly manipulated in experimental settings^42^. Therefore, our findings allow us to interpret the functional significance of the face-associated hands. Fourth, although our PCA results showing three dimensions of grief symptoms appear to be in line with a previously reported three-factor model of ICG-measured grief symptoms, which was derived from a sample of 782 bereaved adults^37^, our PCA results and their associations to behavioral and neural empathic responses should be interpreted cautiously because of the small sample size. Fifth, related to the issue of sample size, we cannot rule out the possibility that the third common variable might have influenced our results because of the heterogeneity of the sample due, for instance, to the different causes and circumstances of the deceased's death among the participants. Further research is needed to replicate the study findings and elucidate whether the within-individual variation in empathy (induced by the current experiments) resembles the way bereaved individuals differently empathize with others across situations in their everyday life, and also how such variation in empathy interferes with the recovery from prolonged grief.

In conclusion, our exploratory findings provide novel insights into the association between grief and empathy for pain that varies under the influence of priming social stimuli; individuals’ grief severity enhanced the expression of empathy for the deceased-primed pain, but it attenuated the brain’s response in the dACC to observing living- and stranger-primed pain. Moreover, our preliminary findings suggested that these associations appeared to be driven by the different aspects of grief presentation: the most major grief dimension representing “longing” interacted with a deceased-related stimulus to enhance empathy, while another grief dimension representing “loss-related avoidance” interacted with a stranger-related stimulus to attenuate the brain’s response to pain, with the latter association being more influential on the grief–empathy relationship.

## Methods

### Participants

We examined our hypotheses using a sample of 28 bereaved adults. Adults aged 20–69 years, whose first language was Japanese, were eligible to participate if they had been bereaved of a relative (spouse, child, parent, sibling, or grandparent) for longer than 12 months before their participation. Participants were recruited from (1) a cohort of bereaved adults who had been enrolled in a clinical therapeutic trial, (2) a self-help group of crime or traffic accident victims, or (3) the general population who responded to advertisements. Psychiatrists screened eligible individuals using the Mini-International Neuropsychiatric Interview^43^. Those screened were excluded if they were currently clinically diagnosed with psychiatric disorders based on the DSM-5, except for depressive disorders, or if they had PTSD, met the criteria for substance dependence in the past 3 months, exhibited severe suicidality in the past 6 months, were pregnant or breast-feeding women, or had a medical condition that could interfere with the study or the interpretation of its results. This study was approved by the Ethics Committee of the National Center of Neurology and Psychiatry (approval number: A2011-015). All participants provided written informed consent prior to study participation and were paid for their participation. All procedures were performed in accordance with the latest Declaration of Helsinki.

### Measures

Participants’ grief severity was determined using the Inventory of Complicated Grief (ICG), a 19-item self-report questionnaire that was well validated with prior evidence for good internal consistency (Cronbach’s α = 0.94) and test–retest reliability (0.80)^23^. Each item is rated on a five-point scale with responses ranging from 0 (*never*) to 4 (*always*), with higher scores indicating increased grief symptoms. The Beck Depression Inventory-II (BDI-II)^44^ and Impact of Event Scale-Revised (IES-R)^45^ were used to determine the extent of depression and posttraumatic stress symptoms, respectively. Violent and sudden deaths were determined according to the prior definition of death events^46^.

### Materials

#### Facial stimuli

Prior to the experiments, each participant provided two portraits for the experiments. One was a portrait of their deceased relative, whose death had caused them grief, serving as the deceased priming face condition (DEC). The other was a portrait of their living relative that the participant deemed to have a similar relationship as they did with the deceased, serving as the living priming face condition (LIV). The two relatives were one of their spouse, offspring, parent, sibling, or grandparent, and both were familiar with each participant, yet different in that they were dead or alive.

In addition, we prepared two portraits. One was a portrait of a stranger, representing a person unfamiliar to the participants, which served as the stranger priming face condition (STR). The other was another stranger with a mosaic face (MOS), serving as the control priming face condition. The color of each photo was converted to grayscale, and the background was replaced with a black color.

#### Pain stimuli

Visual pain stimuli consisted of a series of 36 digital color photos showing pain inflicted by needle injection to the left and right hands of three men and three women^47^. The needle was placed on one of the fingers or the back of the hand with the apparent compression and displacement of the skin around the injected area. In addition, the same constitution of non-painful stimuli as with the painful stimuli was used, in which the needle was covered with a plastic cap and placed next to the finger. Each hand was placed on a black uniform background to highlight the needle and presented in a third-person perspective. These facial and pain stimuli were edited using Adobe Photoshop (Adobe Systems Inc., San Jose, CA).

### Behavioral/fMRI tasks

Behavioral/fMRI experiments were performed between 14:00 and 17:00 for all participants. Tasks were run on a personal computer using E-Prime experimental software (Psychology Software Tools, Pittsburgh, PA). The display was projected onto a screen in the magnet bore, and participants viewed the images via a mirror mounted on the head coil during scanning.

#### Face–empathy task

The purpose of this task was to examine whether an individual’s grief level had different effects on behavioral and neural consequences of observing another’s pain depending on the priming face conditions. Prior evidence indicates that the closeness of the social relationship with an individual could influence the extent to which one would express empathy to that individual (closer relationship, greater empathy)^12–14^. In addition, it is clinically known that bereaved individuals with prolonged grief feel disconnected from their surroundings after the loss^23,24^, suggesting the reduced ability to empathize with close others (living relatives in particular). Therefore, we predicted that participants with higher grief levels would rate the pain stimuli primed with the DEC face as more painful, but would rate the pain stimuli primed with the LIV face as less painful, similar to the STR face. We measured participants’ neural responses to pain ratings immediately after subliminally presenting the faces. We used the subliminal, rather than supraliminal, priming paradigm, as we focused on state empathy that would be expressed more automatically and differentially according to situational factors (e.g., social stimulus type). This paradigm not only prevented participants from altering their pain ratings depending on any specific type of priming stimulus (e.g., DEC face) but also minimized participants’ grief responses to the reminder of their deceased during the experiments.

Participants underwent pain stimulus trials, each of which consisted of the following steps: (1) a brief presentation of a face under any of the four conditions (DEC, LIV, STR, or MOS) for 13 ms^48^; (2) a presentation of another mosaic face as backward masking for 487 ms; and (3) a presentation of the painful or non-painful injected hand stimulus for 1300 ms, This was followed by (4) a blank presentation for 200 ms, and (5) a pain-rating period of 3800 ms. During the pain rating period, each participant was asked to rate the perceived pain intensity in response to each face-primed pain stimulation, using a four-point Likert scale (from “*no pain*” to “*worst pain*”), according to the question “How much does it hurt?” to determine the affective consequence of pain stimulation^47^. The inter-trial interval was 8 s. The faces and pain stimuli were presented in a sequence that was randomized within and across participants. The face–empathy task consisted of 64 trials (4 priming face conditions × 2 pain conditions × 8 trials). The internal consistency of pain ratings was considered good (Cronbach’s α = 0.95).

#### Face task

The purpose of this task was to confirm that the facial stimuli subliminally presented in the aforementioned face–empathy task would not individually activate the known pain empathy networks. Further details are provided in the Supplementary material.

#### Post-scan detection task

The purpose of this task was to confirm that participants were not able to accurately distinguish between the two priming face conditions (i.e., DEC and LIV) subliminally presented during the above two tasks. After completing the MRI scans, participants were asked to report if they had recognized the masked faces presented during the above two tasks and then performed a detection task outside the scanner, in which they were asked to detect the DEC or LIV faces among the four possible masked faces. Further details are provided in the Supplementary material.

### Image acquisition and preprocessing

The parameters for MRI data acquisition and preprocessing steps are provided in the Supplementary material.

### Data analysis

#### Behavior

The interaction effects of grief severity and the priming condition on pain ratings were tested using ANOVA in a general linear model (GLM), with the mean pain ratings across the trials in DEC, LIV, and STR as the dependent variables, the priming condition as a within-subjects factor (three levels) and individuals’ grief severity as a continuous predictor. Univariate tests were used to evaluate the effects of grief severity on pain ratings in each priming condition. The independent effect of grief severity on pain ratings was tested using linear multivariable regression with the ICG scores as the predictor and age, sex, depression (BDI-II), posttraumatic stress symptoms (IES-R), and violent/sudden death as the covariates. Variance inflation factor (VIF) values obtained to identify multicollinearity between the predictors in the model ranged between 1.03 and 3.22, which is generally considered to be acceptable^49^. The significance of the effects was calculated using the likelihood ratio statistic, which provides the most asymptotically efficient test known. For the face task, the possible differences in mean response time or response accuracy were tested between the DEC and LIV conditions. Analyses were performed using commercially available software (StatSoft Statistica 12, Tulsa, OK, USA) and following standard computational procedures. A *p*-value < 0.05 was considered significant.

#### fMRI

The hemodynamic responses were modeled on an event-by-event basis in a GLM design matrix comprising multiple regressors, each of which models the hypothetical hemodynamic time course with the onset of the event stimuli in each condition (DEC-painful, DEC-nonpainful, LIV-painful, LIV-nonpainful, STR-painful, and STR-nonpainful, MOS-painful, MOS-nonpainful) convolved during each event duration (six seconds) with a canonical hemodynamic response function. A high-pass filter was modeled with a cut-off of 1/128 Hz, and serial autocorrelations were modeled as an AR (1) process. Parameter estimates for all the regressors in the GLM (including rigid motions, finite impulse response regressors representing artifacts detected by ART software, and high-pass filter regressors) were obtained voxel-by-voxel by maximum-likelihood estimation. The voxel-by-voxel parameter estimates (beta) in each individual produced a 3D beta image for each parameter, which was fed into the second individual level in the between-subject analyses.

In the first-level analysis, we created two contrasts by comparing the DEC face condition with the MOS face condition and the LIV face condition with the MOS face condition. In the second-level analysis, we first defined the regions specifically engaged in the processing of pain empathy by comparing the hemodynamic responses to pain stimulation with those to non-painful stimuli across all the priming conditions (i.e., DEC, LIV, STR, or MOS). We considered the identified cluster as the “pain empathy region” and created an inclusive binary mask centered on that certified region as the region of interest (ROI). Within this ROI, we tested the effect of grief severity on neural activation separately for the DEC (vs. MOS), LIV (vs. MOS), or STR (vs. MOS) priming conditions, using multiple regression, considering whole-brain activation in DEC, LIV, or STR as the dependent variable and the ICG total score as the independent variable. For the face task, we performed the same preprocessing procedure as that used for the face–empathy task. We compared the neural response to the DEC or LIV faces with that of MOS faces (*t*-test) to confirm whether any known pain empathy regions were engaged specifically in processing the DEC or LIV faces. Activations were corrected for multiple comparisons using familywise error (FWE). Whole-brain analysis inferences were made at the cluster level (*p*_FWE_ < 0.05), based on an initial cluster-forming threshold of *p* < 0.005 (uncorrected)^50^. We accounted for the effects of covariates that could influence neural activation: age, sex, depression (BDI-II score), posttraumatic stress symptoms (IES-R score), and violent/sudden death. To extract parameter estimates across the voxels in a cluster, we used the MarsBaR toolbox (http://marsbar.sourceforge.net/). The automated anatomical labeling in xjView (http://www.alivelearn.net/xjview/) was used to specify the anatomical brain regions of the clusters.

#### Association between grief dimensions and neurobehavioral measures of empathy

To gain further insight into which grief components are more closely associated with pain ratings and related neural responses than the others, we conducted preliminary analyses. First, we produced fewer dimensions of grief symptoms using PCA for individual ICG item scores. The significant components were extracted using Krzanowski cross-validation while striking a balance between model complexity and the ability to accurately predict the data in the presence of PCs’ eigenvalues > 1, and the extracted component scores were then used as predictors to test the effects of grief dimensions on the identified empathy variables (i.e., pain ratings in DEC, extracted neural response in LIV, and extracted neural response in STR) using ANOVA. Two models were run separately for the behavioral and neural empathy variables. Finally, we tested for a general association between the grief dimension variables (i.e., first PC, third PC) on one side and the behavioral/neural empathy variables on the other (i.e., pain ratings in DEC, extracted neural response in LIV, and extracted neural response in STR), using canonical correlation analysis (CCA). CCA, given two sets of variables (X and Y), finds a linear combination of X that is maximally correlated with a linear combination of Y (i.e., a weighted sum of each variable). It thus creates pairs of canonical variates that explain the relationship between X and Y. For each pair of canonical variates, we can determine the contribution of each variable to the opposite canonical variate, and thus the strength and direction of the relationships between each variable and the opposite set of variables^51^. The VIF values within variable sets ranged between 1.00 and 1.83, suggesting that multicollinearity was not an issue^49^. Analyses were performed using StatSoft Statistica 12 (Tulsa, OK, USA) and a *p*-value < 0.05 was considered significant.

## Supplementary Information

Supplementary Information.

## Data Availability

The data that support the findings of this study are available from the corresponding author upon reasonable request.
