# Supplementary material for: Exploring the role of empathy in prolonged grief reactions to bereavement
Source: Sci Rep. 2023 May 10;13:7596. doi: 10.1038/s41598-023-34755-y (PMC10172345; doi:10.1038/s41598-023-34755-y)
Supplement: Supplementary file 1 — Supplementary Information. [file 41598_2023_34755_MOESM1_ESM.docx]

**Supplementary Information**

**Exploring the role of empathy in prolonged grief reactions to bereavement**

**Supplementary Methods**

**Face task**

The purpose of this task was to confirm that the same facial stimuli presented in the face–empathy task would not individually activate the known pain empathy networks. In this task, we measured participants’ neural responses to a brief presentation of either of the four faces, each followed by a simple response trial. Participants were asked to press a button as quickly as possible each time an alphabetical character appeared. The mean reaction time and response rates were also determined. Each trial consisted of the following steps: (1) and (2) were the same as those in the face–empathy task; 3) a presentation of a blank screen for a jittered period (100–500 ms), followed by 4) presentation of any one of the 26 alphabetical characters for 500 ms. The inter-trial interval was six seconds. The faces and characters were presented in a sequence that was randomized within and across participants. The face task consisted of 64 trials (four face conditions × 16 trials).

**Post-scan detection task**

Given that participants had been informed prior to the experiment that two photos (i.e., DEC and LIV) would be used in any form during the experiment, the participants’ expectancy for the presentation of photos could influence their post-scan report of their own awareness (i.e., participants would tend to report that they saw the DEC and/or LIV faces despite not being fully aware of the two). First, outside the scanner, after all MRI scans were performed, we asked the participants to report whether they had recognized the STR face, as well as the DEC and LIV faces, which were briefly presented during the scanning. Unawareness of the STR face, which was unexpectedly presented as the “dummy” condition, would support the lack of awareness of the masked face stimuli even when participants could not help but report awareness of the known DEC and/or LIV faces presented during the scan.

We then employed an objective measure of awareness to confirm that the participants were not explicitly aware of the masked DEC and LIV faces and could not accurately detect differences between the DEC face and the other three (LIV, MOS, and STR) or between the LIV face and the other three (DEC, MOS, and STR). Participants were informed that immediately before the mosaic face, another face would appear so briefly that it would be difficult to perceive, and that on each trial they could recognize one of four possible types of masked face (i.e., DEC, LIV, MOS, or STR). This task consisted of 40 trials of presentation of the same face stimuli as those used in the tasks during the scan (4 face conditions × 10 trials). Each trial consisted of the following steps: steps 1) and 2) were the same as those in the face–empathy task; 3) participants were then asked to indicate when the DEC or LIV face was presented by pressing either of two buttons (e.g., button “1” for the DEC and button “2” for the LIV). Using signal detection theory, discriminability (*d*’) was calculated for the DEC and LIV conditions, considering the hit and false alarm rates, with *d*’ = 0 indicating chance performance. We also calculated the rate of button pressing across the entire session to confirm whether participants responded similarly to the stimuli. We predicted that the detection performance would not significantly exceed the chance level and that the DEC and LIV faces would not differentially affect the face detection ability.

**Image acquisition and preprocessing**

Gradient echo-planar images (EPIs) were acquired on a 3.0-Tesla scanner (MAGNETOM Verio 3T, Siemens, Germany) using a 12-channel head coil. For each functional run, 256 and 192 T2*-weighted axial slices, parallel to the AC–PC plane, were acquired for the face–empathy task and face task, respectively, using an EPI pulse sequence (TR = 2000 ms, TE = 30 ms, flip angle = 90°, field of view = 256 mm, number of slices = 36, slice thickness = 3 mm, matrix size = 64 × 64). Three dummy scans were performed before the fMRI acquisition to obtain longitudinal magnetization equilibrium. The total acquisition time was 8 min 56 s for the face–empathy task and 6 min 48 s for the face task.

Images were computed, overlaid on anatomical images, and analyzed using Statistical Parametric Mapping software (SPM12, Wellcome Department of Imaging Neuroscience, Institute of Neurology, and the National Hospital for Neurology and Neurosurgery; London, England), implemented in MATLAB R2017a (Mathworks, Inc., Sherborn, MA). fMRI scans were preprocessed using the following procedure: First, slice timing correction was performed to reduce mismatching between the acquisition timing of different slices; all images were realigned to the first image as a reference to minimize variance due to head movements. In addition, functional images were co-registered to the individual structural T1-weighted images, which were then spatially normalized to a standard T1 template volume image based on the Montreal Neurological Institute (MNI) reference brain. The normalization parameters were then applied to all functional images, which enabled a more precise comparison between individuals, with the output images resliced into a voxel size of 3 × 3 × 3 mm. Finally, smoothing was applied, using a 6 mm full-width at half-maximum (FWHM) isotropic Gaussian kernel, with the purpose of removing high-frequency information and enhancing the signal-to-noise ratio. Data were corrected for rigid body motion artifacts using the Artifact Detection Tools (ART; https://www.nitrc.org/projects/artifact_detect/) toolbox.

**Supplementary Results**

**Face task**

As expected, there was no statistically significant difference in response time between DEC and LIV faces (DEC: 332.9 ± 44.5 ms; LIV: 325.9 ± 42.3 ms; *t*_54_ = 0.60, *p* = .54) or response accuracy (DEC: 92.2 ± 14.4%; LIV: 90.4 ± 14.8%; *t*_54_ = 0.46, *p* = .65), suggesting that the subliminal presentation of DEC or LIV faces in the face–empathy task did not differentially affect participants’ sustained attention. In line with these behavioral results, the subliminal DEC or LIV faces themselves did not individually influence the known pain empathy networks, as there were no significant differences in neural activation in the DEC (*p*_FWE_ > .71) or LIV (*p*_FWE_ > .44) conditions compared to that in the MOS condition.

**Post-scan detection task**

After being informed about the briefly presented faces during the scanner experiment, eight participants (28.6%) reported that they had recognized one or both of the faces (i.e., DEC or LIV) in the photographs that they had provided. As predicted, however, no one noticed the STR face that we prepared, suggesting a lack of awareness of the masked face stimuli presented without any prior knowledge of these. The rate of button presses during the task was 31.4 ± 0.25% on average across participants. As predicted, the average *d*’ value did not differ significantly from 0, for either the DEC (*p* =1.00) or LIV faces (*p* =1.00), indicating that even if the patients reported the awareness of one or both of the DEC and LIV faces, they were unable to accurately detect the DEC or LIV faces among the presented faces. Additionally, the above results (low average *d*’) were unaffected by masked faces (*F*_1, 26_ = 0.73, *p* =.40, *η*^2^ = 0.03), grief levels (*F*_1, 26_ = 1.67, *p* = .21, *η*^2^ = 0.06), or face × grief interaction (*F*_1, 26_ = 1.04, *p* = .32, *η*^2^ = 0.039).

**
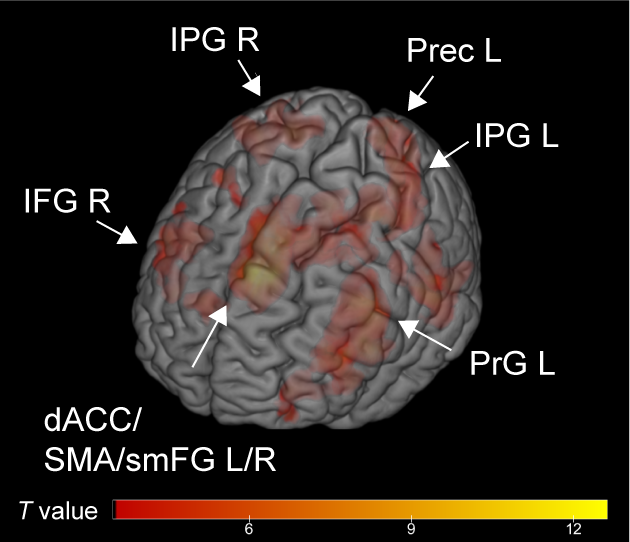
**

**Figure S1.** A three-dimensional reconstruction of the differences in activation between pain conditions (painful > non-painful). Regions in which participants had greater activation in the painful condition compared to the non-painful condition across the four priming face conditions are shown in red/yellow with T values. Clusters shown are false discovery rate whole-brain corrected at *p* < .05.

Abbreviations: dACC, dorsal anterior cingulate cortex; IFG, inferior frontal gyrus; IPG, inferior parietal gyrus; L, left; Prec, Precuneus; PrG, precentral gyrus; R, right; SMA, supplementary motor area; smFG, superior medial frontal gyrus.

**Table S1. Brain Areas that Demonstrate Greater Hemodynamic Response to Painful than Non-painful Stimulations**

|  |  |  | MNI coordinates | | |  |  |  | Cluster |
| --- | --- | --- | --- | --- | --- | --- | --- | --- | --- |
| Brain area | Hem | BA | *x* | *y* | *z* | *T* | *Z* | *p*_FWE_ | *k* |
| Superior medial frontal gyrus | L | 24 | 0 | 27 | 39 | 13.86 | 7.01 | < .0001 | 3448 |
| Middle cingulate gyrus | L | 32 | –6 | 21 | 36 | 11.19 | 6.41 |  |  |
| Supplementary motor area | L | 32 | –3 | 18 | 45 | 9.16 | 5.82 |  |  |
| Precuneus | L | 7 | –9 | –63 | 54 | 7.14 | 5.08 | < .0001 | 1632 |
| Inferior parietal gyrus | R | 40 | 30 | –51 | 48 | 6.44 | 4.78 |  |  |
| Supramarginal gyrus | L | 48 | –63 | –21 | 27 | 6.30 | 4.72 |  |  |
| Cerebellum, Crus 1 | L |  | –33 | –69 | –30 | 6.82 | 4.95 | < .0001 | 1705 |
| Lingual gyrus | R | 17 | 3 | –78 | –6 | 6.71 | 4.90 |  |  |
| Fusiform gyrus | R | 19 | 27 | –78 | –12 | 6.29 | 4.71 |  |  |
| Pallidum | L | 25 | –12 | 6 | –6 | 6.65 | 4.87 | .029 | 97 |
| Pallidum | L |  | –15 | 6 | 6 | 4.28 | 3.61 |  |  |
| Thalamus | R |  | 6 | –3 | 6 | 4.15 | 3.53 |  |  |

Clusters shown are familywise error (FWE) corrected for multiple comparisons at *p* < .05.

Abbreviations: BA, Brodmann area; Hem, hemisphere; L, left; MNI, Montreal Neurological Institute; R, right.

**Table S2. Loadings of Extracted Principal Components for ICG Item Scores**

|  | Principal Component | | |
| --- | --- | --- | --- |
| ICG Item | 1st | 2nd | 3rd |
| 1. Preoccupation with the deceased | **0.75** | –0.22 | 0.04 |
| 2. Memories of the deceased upset me | **0.87** | 0.17 | –0.11 |
| 3. Nonacceptance of the death | **0.75** | –0.28 | 0.11 |
| 4. Longing for the deceased | **0.90** | 0.03 | -0.22 |
| 5. Drawnness to places or things related to the deceased | **0.42** | 0.36 | 0.23 |
| 6. Anger about the death | **0.76** | 0.23 | 0.05 |
| 7. Disbelief over what happened | **0.73** | –0.21 | 0.13 |
| 8. Feeling stunned or dazed | **0.79** | –0.17 | 0.29 |
| 9. Difficulty trusting people | **0.74** | –0.27 | 0.15 |
| 10. Feeling distant from people | **0.73** | **–0.47** | 0.22 |
| 11. Physical pain | **0.56** | **0.66** | 0.11 |
| 12. Avoidance of reminders of the deceased | **0.57** | **0.40** | **–0.49** |
| 13. Emptiness without the deceased | **0.81** | –0.26 | 0.11 |
| 14. Auditory Hallucination | 0.30 | **0.71** | 0.18 |
| 15. Visual Hallucination | **0.46** | **0.52** | 0.33 |
| 16. Feeling unfair that I should live when the person died | **0.73** | –0.04 | 0.37 |
| 17. Bitterness over the death | **0.84** | –0.13 | **–0.40** |
| 18. Feeling envious of others who have not lost someone close | **0.70** | –0.03 | **–0.42** |
| 19. Loneliness since the loss | **0.84** | 0.01 | –0.34 |

Loadings greater than |0.40| are shown in bold.

Abbreviation: ICG, Inventory of Complicated Grief.
